# PPARγ activation by troglitazone enhances human lung cancer cells to TRAIL-induced apoptosis via autophagy flux

**DOI:** 10.18632/oncotarget.15819

**Published:** 2017-03-01

**Authors:** Uddin MD. Nazim, Ji-Hong Moon, You-Jin Lee, Jae-Won Seol, Sang-Youel Park

**Affiliations:** ^1^ Biosafety Research Institute, College of Veterinary Medicine, Chonbuk National University, Iksan, Jeonbuk 54596, South Korea

**Keywords:** troglitazone, PPARγ, autophagy, TRAIL, lung cancer cells

## Abstract

Members of the tumor necrosis factor (TNF) transmembrane cytokine superfamily, such as TNFα and Fas ligand (FasL), play crucial roles in inflammation and immunity. TRAIL is a member of this superfamily with the ability to selectively trigger cancer cell death but does not motive cytotoxicity to most normal cells. Troglitazone are used in the cure of type II diabetes to reduce blood glucose levels and improve the sensitivity of an amount of tissues to insulin. In this study, we revealed that troglitazone could trigger TRAIL-mediated apoptotic cell death in human lung adenocarcinoma cells. Pretreatment of troglitazone induced activation of PPARγ in a dose-dependent manner. In addition conversion of LC3-I to LC3-II and PPARγ was suppressed in the presence of GW9662, a well-characterized PPARγ antagonist. Treatment with troglitazone resulted in a slight increase in conversion rate of LC3-I to LC3-II and significantly decreased p62 expression levels in a dose-dependent manner. This indicates that troglitazone induced autophagy flux activation in human lung cancer cells. Inhibition of autophagy flux applying a specific inhibitor and genetically modified ATG5 siRNA enclosed troglitazone-mediated enhancing effect of TRAIL. These data demonstrated that activation of PPARγ mediated by troglitazone enhances human lung cancer cells to TRAIL-induced apoptosis via autophagy flux and also suggest that troglitazone may be a combination therapeutic target with TRAIL protein in TRAIL-resistant cancer cells.

## INTRODUCTION

Members of the tumor necrosis factor transmembrane cytokine superfamily, such as TNFα and Fas ligand (FasL), play crucial roles in inflammation and immunity [1]. TRAIL is a member of this superfamily with the ability to selectively trigger cancer cell death, but does not motive cytotoxicity to most normal cells [2]. Five members of the death receptor family have been established that can bind TRAIL. The death receptors, DR4 (TRAIL-R1) and DR5 (TRAIL-R2), involve two cysteine-rich extracellular TRAIL-mandatory domains and a cytoplasmic death domain, which are essential for transmitting a cytotoxic signal [3, 4]. The decoy receptors, DcR1 (TRAIL-R3), DcR2 (TRAIL-R4), and osteoprotegerin (TRAIL-R5), can also bind TRAIL, but they do not transmit apoptotic signals as a result of a nonfunctional death domain [5, 6]. Finally, TRAIL initiates apoptosis upon the binding of TRAIL receptors, which promotes the recruitment of the accessory molecule FAS-associated death domain protein and ultimately, procaspase-8, to the formation of death-inducing signaling complex, leading to subsequent effector caspases (caspase-8, -9, -10, and -3) [7, 8]. However, the downregulation of DR, upregulation of anti-apoptotic protein, and ultimately, upregulation of inhibitors of apoptosis proteins contributes resistance to TRAIL-mediated apoptosis in many cancer cells [9–13]. Many studies have demonstrated that the related mechanisms of association exist between TRAIL and numerous agents [14–23]. Accordingly, combination treatment with TRAIL sensitizers is one direction to overcoming TRAIL resistance.

The peroxisome proliferator-activator receptors are members of ligand-stimulated nuclear receptors family that include PPARα, PPARδ, and PPARγ [24]. PPARγ is mainly exposed in adipose tissue and plays eventual roles in lipid metabolism, adipocyte differentiation, and progressing insulin sensitivity [25]. Notwithstanding, information on the molecular mechanism of PPARγ in tumorigenesis is fractioning. Many studies have revealed that PPARγ acts as a tumor inhibitor, because it is mainly exposed in prostate, breast, and colonic epithelium and the appearance of cultured cancer cell lines suppositious of these tissue types with ligands of PPARγ restrain cellular proliferation [26–31].

Fortunately, many studies have revealed that chemotherapeutic drugs can lead to increased apoptosis induction in TRAIL-resistant tumor cells [32, 33]. The thiazolidinedione (TZD) groups of antidiabetic drugs, such as troglitazone are used in the cure of type II diabetes to reduce blood glucose levels and improve the sensitivity of numerous tissues to insulin [34]. The ability of TZDs to inhibit a number of cancers has been associated with their ability to suppress growth and stimulate apoptosis in a PPARγ-dependent manner [35, 36]. One recent study has shown that the activation of PPARγ induces autophagy in breast cancer cells [37].

Autophagy has been characterized as a particular homeostatic and adaptive system that serves to convey cytoplasmic components and organelles to the lysosomes for digestion [38, 39]. The autophagy process starts with the formation of double-membrane vesicles conversant as autophagosomes that engulf cytoplasmic constituents including organelles proceeding from a maturation system upon fusion with lysosomes and finally, dilapidation and recycling of the cargo [40, 41]. Autophagosome formation is coordinated by the Atg12-Atg5-Atg16 complex and conversion of LC3-I-phospholipid conjugates LC3-II, which is used extensively as an autophagy marker [42, 43]. P62 is a well-establish autophagy marker that is organized into autophagosomes by exactly interacting with LC3 and is comfortably degraded by autophagy. Autophagy and apoptosis coordinately govern cell survival and cell death, and appear simultaneously in cancers [44, 45]. Chemotherapeutic agents frequently activate autophagy in cancer cells. Therefore, activation of autophagy in cancer cells has been revealed to play an extensive induction as a cell death mechanism by tumor development, where apoptosis is limited [46].

Though the effect of the antidiabetic drugs such as troglitazone and the synergistic outcome of troglitazone combined with TRAIL are well-establish; however, the molecular mechanisms involved in these effects are presently unknown. Therefore, the objective of this research was to supervise the molecular mechanisms underlying the anticancer effect of troglitazone and the synergistic outcome of troglitazone combined with TRAIL in lung A549 cells.

## RESULTS

### Troglitazone sensitizes TRAIL-mediated apoptosis in human lung adenocarcinoma cells

**Figure 1:**
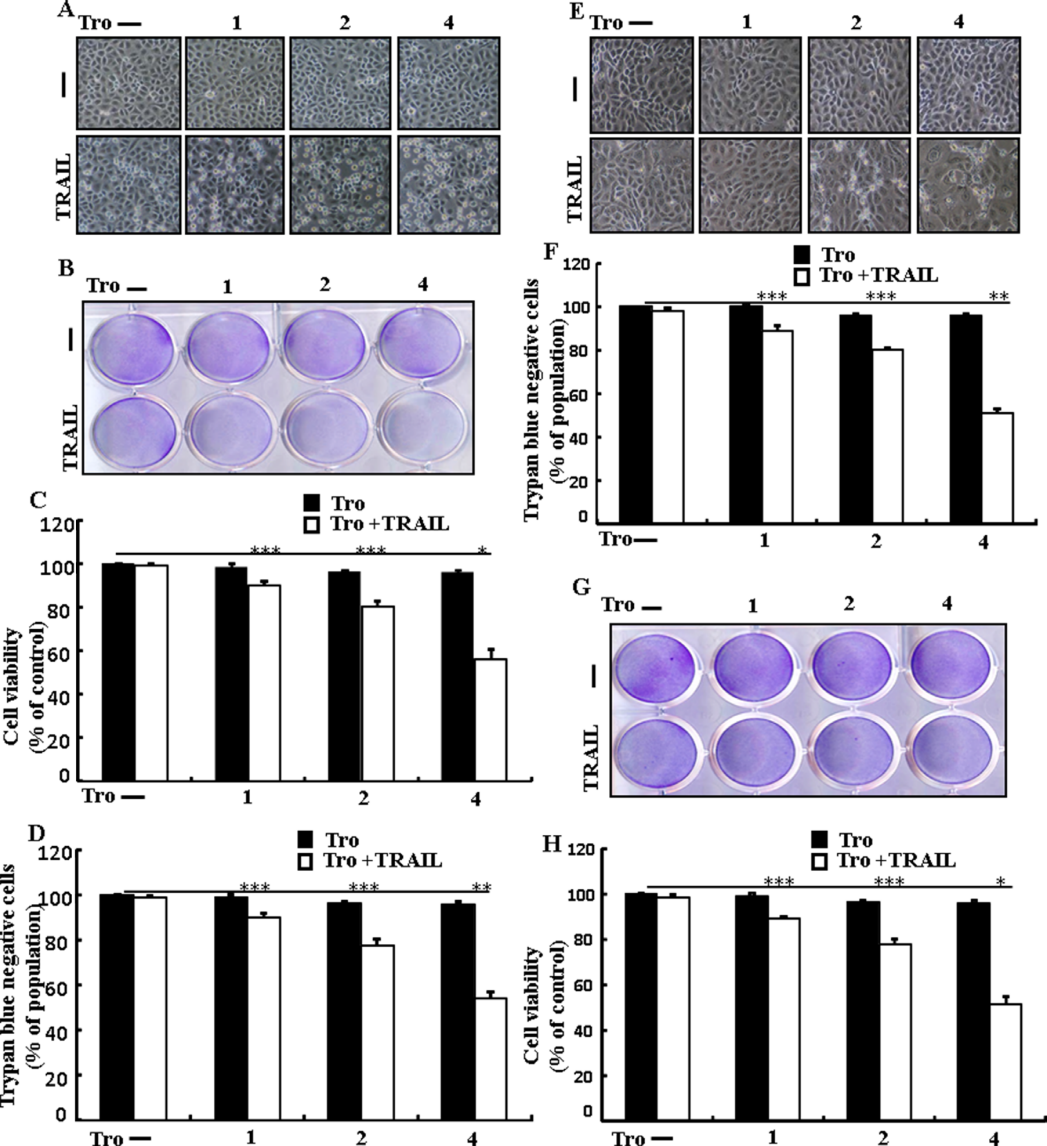
Troglitazone sensitizes TRAIL-mediated apoptosis in human lung adenocarcinoma cells A549, HCC-15 and Calu-3 cells were pre-incubated with troglitazone at different doses (0, 1, 2, and 4 μM) for 12 h and exposed to TRAIL protein 200 ng/ml for 2 h. (**A** and **E**) Cell morphology photographed using light microscope in A549 and Calu-3 Cells (×100); (**B** and **G**) Cell viability was measured with crystal violet assay in A549 and HCC-15 Cells; (**C** and **H**) Bar graph indicating the average density of crystal violet in A549 and HCC-15 Cells; (**D** and **F**) Cell viability was measured with trypan blue dye exclusion assays in A549 and Calu-3 Cells. *p < 0.05 **p < 0.01, ***p < 0.001: represent significant differences between control and each treatment group; Tro: Troglitazone; TRAIL: Tumor necrosis factor (TNF)-related apoptosis-inducing ligand.

To understand the effect of troglitazone on TRAIL-mediated apoptosis, lung adenocarcinoma cells were pre-incubated with different concentrations of troglitazone for 12 h and exposed to TRAIL for 2 h. We photographed adenocarcinoma cells under a light microscope to visualize the morphological changes. Treatment of TRAIL or troglitazone alone did not or only slightly induced cell death (Figure 1) and did not morphological change was identified compared with that in control, suggesting that A549 cells were highly resistant to TRAIL-induced apoptosis. Nevertheless, co-treatment of TRAIL with serial concentrations of troglitazone significantly reduced the cell viability compared with troglitazone or TRAIL alone (Figure 1A–1D). Furthermore, troglitazone and TRAIL co-treatment also decreased the cell viability and significantly sensitized apoptosis in Calu-3, HCC-15 cells (Figure 1E–1H). These data suggested that troglitazone sensitized TRAIL-mediated apoptosis in lung adenocarcinoma cells.

### Troglitazone induces autophagy and sensitized apoptosis mediated by TRAIL

**Figure 2:**
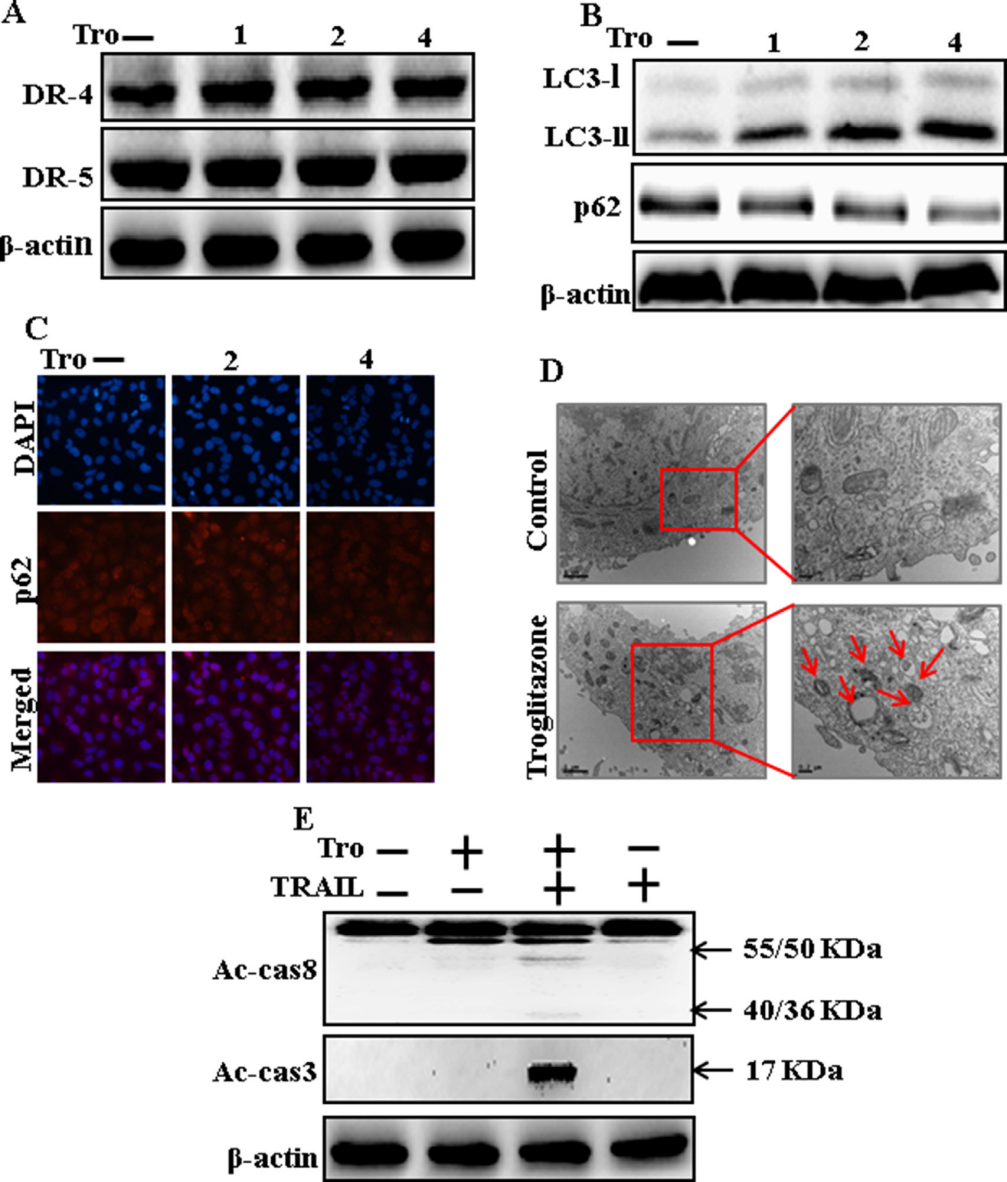
Troglitazone induces autophagy and sensitized apoptosis mediated by TRAIL A549 cells were pre-incubated with troglitazone at varying doses (0, 1, 2, and 4 μM) for 12 h. (**A** and **B**) Western blot for DR-4, DR-5, LC3-II, and p62 proteins was analyzed from A549 cells; (**C**) Cells were immunostained with p62 antibody (red) and observed in fluorescent view; (**D**) TEM shows the ultrastructure of cells treated with troglitazone for 12 h. Arrows indicate autophagosomes, together with residual digested material and empty vacuoles; (**E**) Western blot for Ac-cas3 and Ac-cas8 expression levels was conducted with A549 cells. Cells were pre-incubated with troglitazone for 12 h and exposed to TRAIL protein for an additional 1 h. β-actin was used as the loading control. Tro: Troglitazone; TRAIL: Tumor necrosis factor (TNF)-related apoptosis-inducing ligand; Ac-cas3: Activated caspase 3; Ac-cas8: Activated caspase 8.

To understand the effect of troglitazone on autophagy flux. All the cell lysates were included to western blot analysis. As displayed in Figure 2A, the protein expression levels of DR4 and DR5, were unchanged by troglitazone at varying concentrations. P62 is a well-establish autophagy marker that is organized into autophagosomes by exactly interacting with LC3 and is comfortably degraded by autophagy. Inhibiting autophagy results in prompt accumulation of cellular p62, on the contrary decreased p62 levels are amalgamated with activating autophagy. However, LC3-II was significantly increased and p62 was decreased after troglitazone treatment in a dose-dependent manner (Figure 2B). Immunocytochemistry results also supported that various concentrations of troglitazone decreased p62 protein levels (Figure 2C). A TEM assay suggested that numerous autophagic vacuoles and empty vacuoles were appeared in the cells treated with troglitazone (Figure 2D). The combined treatment of troglitazone and TRAIL enhanced intracellular apoptosis indicators Ac-cas3 and Ac-cas8 expression levels compare with the single treatment with TRAIL or troglitazone (Figure 2E). These results suggested that troglitazone could induce autophagy in A549 cells.

### Troglitazone enhancement of TRAIL-induced apoptosis is blocked by inhibition of autophagy

**Figure 3:**
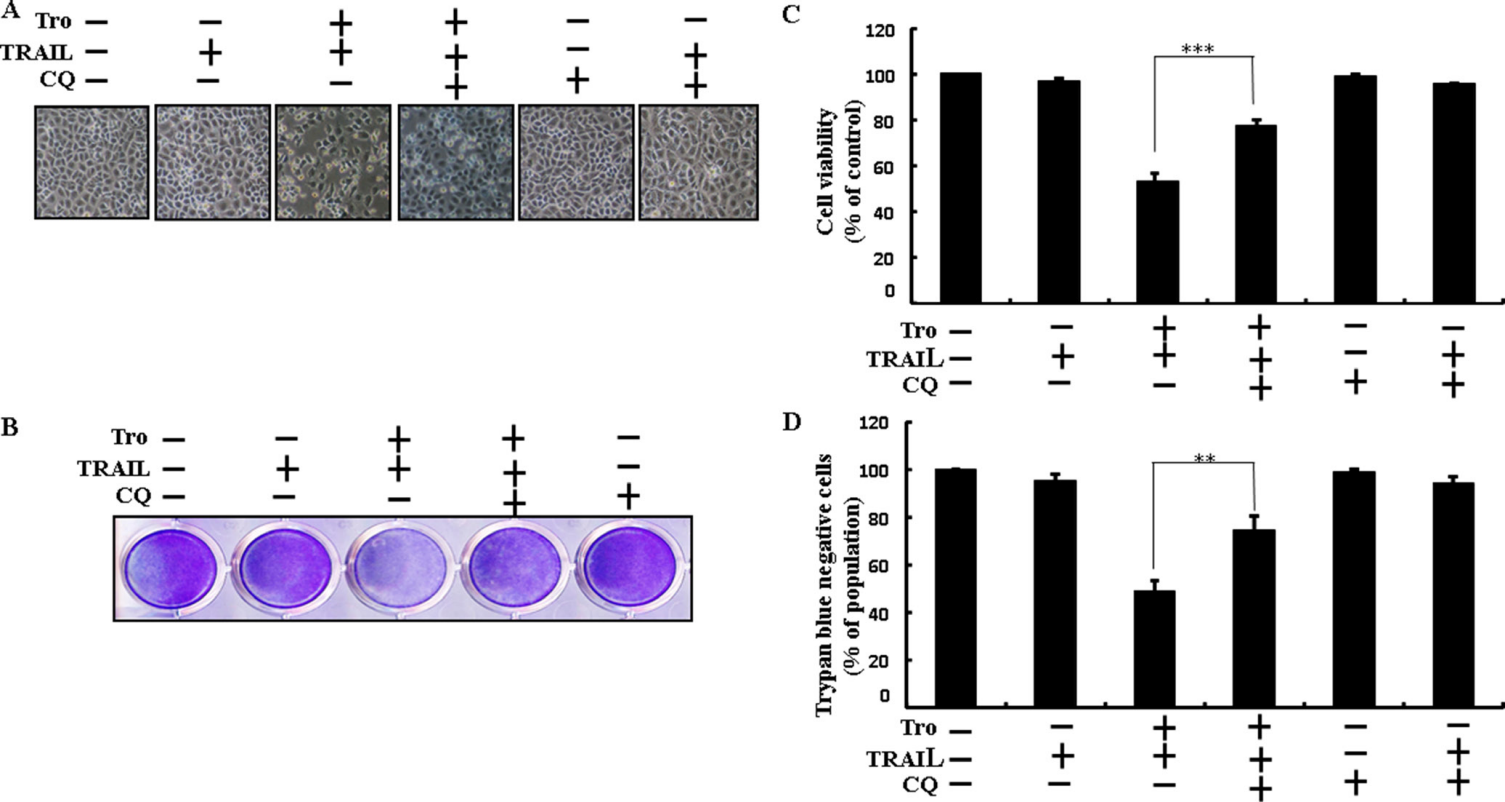
Troglitazone enhancement of TRAIL-induced apoptosis is blocked by inhibition of autophagy Cells were pre-incubated with the indicated troglitazone doses for 12 h and exposed to TRAIL protein for an additional 2h. Additional cells were also pre-incubated with autophagy inhibitor chloroquine for 1 h followed by troglitazone treatment. (**A**) Cell morphology photographed using light microscope (×100); (**B**) Cell viability was measured with crystal violet assay; (**C**) Bar graph indicating average density of crystal violet; (**D**) Cell viability was measured with trypan blue dye exclusion assays. **p < 0.01, ***p < 0.001: represent significant differences between control and each treatment group; Tro: Troglitazone; TRAIL: Tumor necrosis factor (TNF)-related apoptosis-inducing ligand; CQ: Chloroquine.

Chloroquine was used to investigate the effect of troglitazone on TRAIL-induced apoptosis. A549 cells were pre-incubated with the indicated troglitazone concentrations for 12 h and exposed to TRAIL for 2h. A549 cells were also pre-incubated with autophagy inhibitor chloroquine for 1 h followed by troglitazone. Co-treatment of troglitazone, chloroquine, and TRAIL blocked cell death. However, Cell morphology results also supported that chloroquine enclosed the cell death effect compared to treatment with troglitazone and TRAIL (Figure 3A). Co-treatment of troglitazone, TRAIL, and chloroquine strongly increased cell viability in human lung adenocarcinoma A549 cells with significantly decreased cell death (Figure 3B–3D). These data suggested that chloroquine could promote troglitazone-mediated cancer cell survival induced by TRAIL.

### Inhibition of autophagy blocks TRAIL-mediated apoptosis by troglitazone through activation of autophagy flux

**Figure 4:**
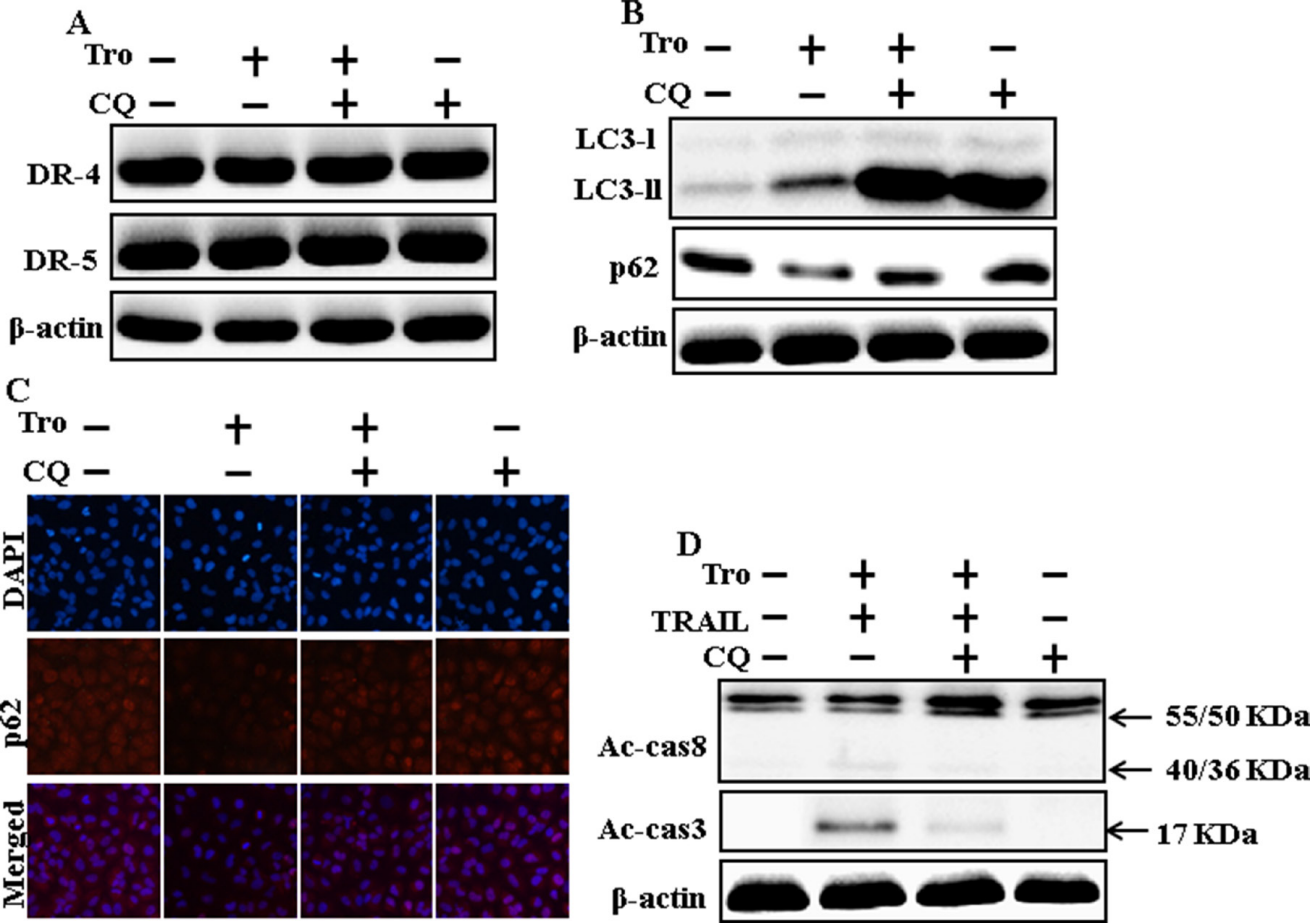
Inhibition of autophagy blocks TRAIL-mediated apoptosis by troglitazone via activation of autophagy flux A549 cells were pre-incubated with chloroquine for 1h followed by indicated troglitazone doses for 12 h. (**A** and **B**) Western blot for DR-4, DR-5, LC3-II, and p62 proteins was analyzed from A549 cells; (**C**) Cells were immunostained with p62 antibody (red) and observed in fluorescent view; (**D**) Western blot for Ac-cas3 and Ac-cas8 expression levels was conducted with A549 cells. Cells were pre-incubated with the indicated troglitazone concentrations for 12 h and exposed to TRAIL protein for an additional 1h. Additional cells were pre-incubated with autophagy inhibitor chloroquine for 1 h, followed by troglitazone treatment. β-actin was used as the loading control. Tro: Troglitazone; Tumor necrosis factor (TNF)-related apoptosis-inducing ligand; Ac-cas3: Activated caspase 3; Ac-cas8: Activated caspase 8; CQ: Chloroquine.

We determine the effect of troglitazone on TRAIL induction of the apoptotic pathway by activating autophagy flux with pharmacological autophagy inhibitor chloroquine. All the cell lysates were included to western blot analysis. The expression levels of DR4 and DR5 were unchanged by troglitazone or chloroquine alone or by combined treatment with troglitazone and chloroquine in A549 cells (Figure 4A). Autophagy induction was further adopted by the observation of autophagic flux using chloroquine. Autophagy inhibitor Chloroquine caused impressed accumulation of membrane-bound LC3-II levels, with decreasing p62 (Figure 4B). Immunocytochemistry results also supported that troglitazone treatment decreased the p62 protein level compared with chloroquine or by treatment with both troglitazone and chloroquine (Figure 4C). The combined treatment of troglitazone and TRAIL enhanced intracellular apoptosis indicators Ac-cas3 and Ac-cas8 expression levels. However, co-treatment of troglitazone, TRAIL, and chloroquine enclosed the increase in expression level of Ac-cas3 and Ac-cas8 (Figure 4D). These results suggested that troglitazone-mediated enhancement of the TRAIL-induced apoptosis could be blocked by chloroquine via activation of autophagy flux.

### Troglitazone enhanced TRAIL-induced apoptosis is blocked by genetic inhibition of autophagy

**Figure 5:**
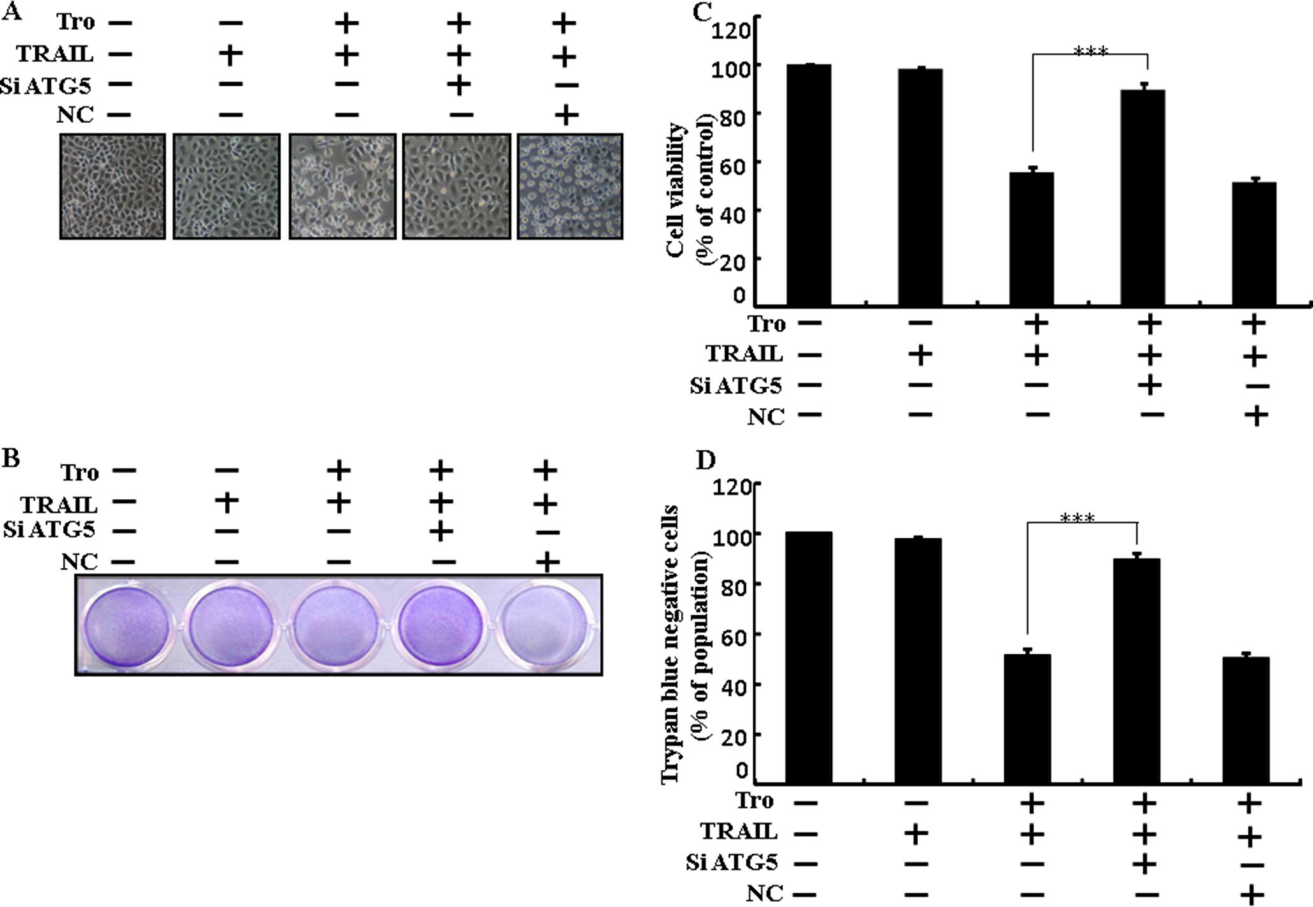
Troglitazone enhanced TRAIL-induced apoptosis is blocked by genetic inhibition of autophagy A549 cells were pre-incubated with ATG5 siRNA or negative control siRNA for 24 h and then exposed to indicated troglitazone doses for 12 h with or without TRAIL protein for an additional 2 h. (**A**) Cell morphology photographed using light microscope (×100); (**B**) Cell viability was measured with crystal violet assay; (**C**) Bar graph indicating average density of crystal violet; (**D**) Cell viability was measured with trypan blue dye exclusion assays. ***p < 0.001: represent significant differences between control and each treatment group. Tro: Troglitazone; TRAIL: Tumor necrosis factor (TNF)-related apoptosis-inducing ligand; siATG5: ATG5 small interfering RNA; NC: Negative control.

Genetic autophagy inhibitor ATG5 siRNA used to determine the effect of troglitazone on TRAIL-induced apoptosis. A549 cells were pre-incubated with ATG5 siRNA or NC for 24 h and then exposed to indicate troglitazone doses for 12 h with or without TRAIL for 2 h. Co-treatment of troglitazone, ATG5 siRNA, and TRAIL blocked cell death. However, Cell morphology results also supported that ATG5 siRNA blocked cell death effect compared to troglitazone, TRAIL, and negative control siRNA treatment (Figure 5A). Co-treatment with troglitazone, TRAIL, and ATG5 siRNA strongly increased cell viability in human lung adenocarcinoma A549 cells with significantly decreased cell death (Figure 5B–5D). These results suggested that ATG5 siRNA could promote troglitazone-mediated cancer cell survival induced by TRAIL.

### Genetic inhibition of autophagy blocks TRAIL-induced apoptosis by troglitazone through activation of autophagy flux

**Figure 6:**
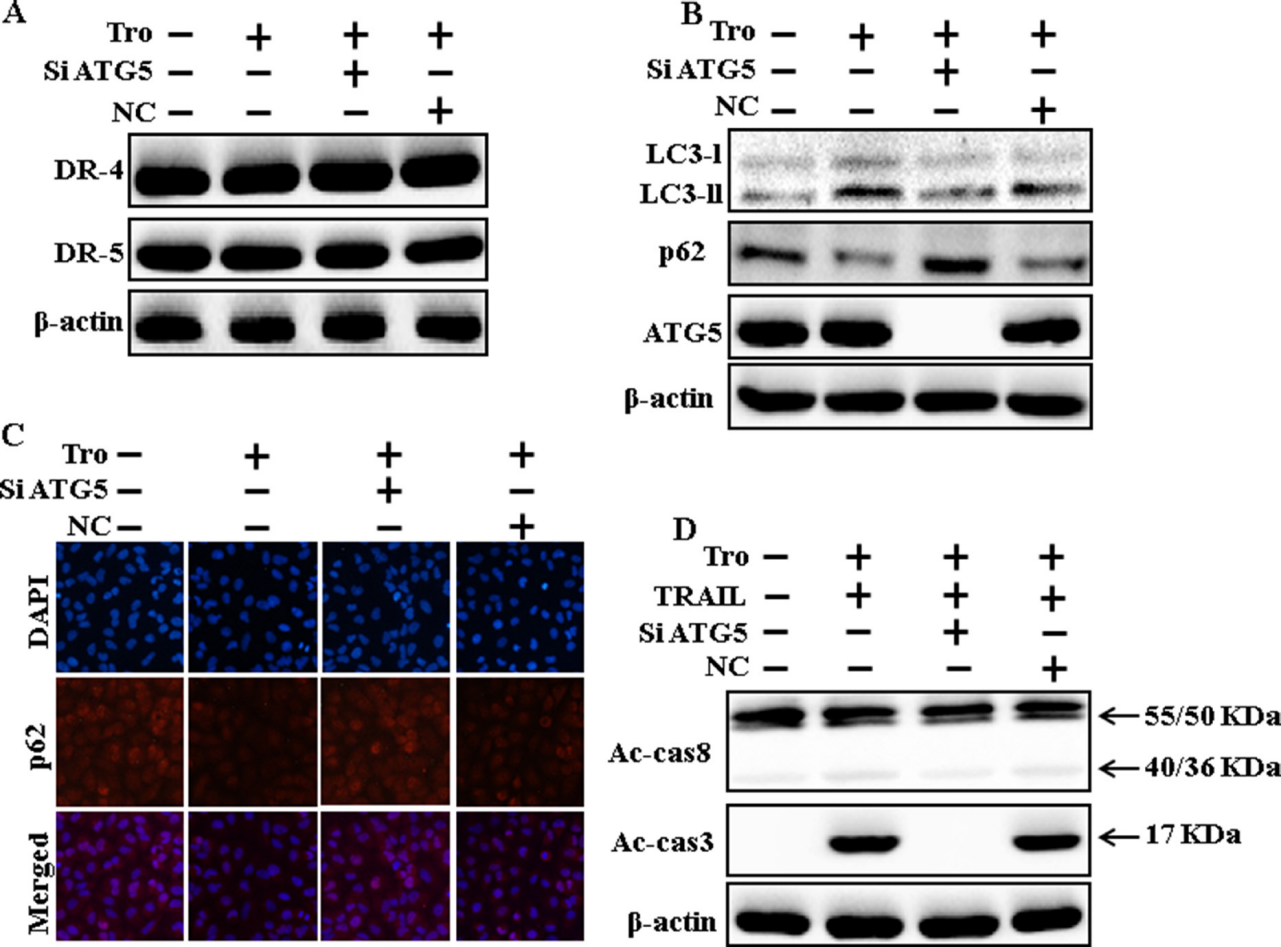
Genetic inhibition of autophagy blocks TRAIL-induced apoptosis by troglitazone via activation of autophagy flux A549 cells were pre-incubated with ATG5siRNA or negative control siRNA for 24 h, and then exposed to indicated troglitazone doses for 12 h. (**A** and **B**) Western blot for DR-4, DR-5, LC3-II, p62 and ATG5 proteins was analyzed from A549 cells; (**C**) Cells were immunostained with p62 antibody (red) and observed in fluorescent view; (**D**) Western blot for Ac-cas3 and Ac-cas8 expression levels was conducted. A549 cells were pre-incubated with ATG5siRNA or negative control siRNA for 24 h, and then exposed to indicated troglitazone doses for 12 h with or without TRAIL protein for an additional 1 h. β-actin was used as the loading control. Tro: Troglitazone; TRAIL: Tumor necrosis factor (TNF)-related apoptosis-inducing ligand; Ac-cas3: Activated caspase 3; Ac-cas8: Activated caspase 8; siATG5: ATG5 small interfering RNA; NC: Negative control.

To understand the effect of troglitazone-induced TRAIL-mediated apoptotic pathway by activating autophagy flux with genetic autophagy inhibition by ATG5 siRNA. All the cell lysates were included to western blot analysis. The expression levels of DR4 and DR5 were unchanged by troglitazone alone or by combined treatment with ATG5 siRNA or NC siRNA in A549 cells (Figure 6A). To address the induction of autophagy, cells were transfected with siRNA directed in opposition to autophagy protein 5 (Atg5) to block autophagic vesicle composition, and silencing of ATG5 was confirmed. Knockdown of ATG5 markedly decreased the troglitazone-induced LC3-II protein level (Figure 6B). Immunocytochemistry results also suggested this p62 protein level in A549 cells (Figure 6C). Co-treatment of troglitazone, NC siRNA, and TRAIL enhanced intracellular apoptosis indicators Ac-cas3 and Ac-cas8. However, co-treatment with troglitazone, ATG5 siRNA, and TRAIL enclosed the increase in Ac-cas3 and Ac-cas8 expression levels (Figure 6D). These results suggested that troglitazone-mediated enhancement of the TRAIL-induced apoptosis could be blocked by genetic inhibition of autophagy via activation of autophagy flux.

### Effects of troglitazone are induced by PPARγ activation in A549 cells

**Figure 7:**
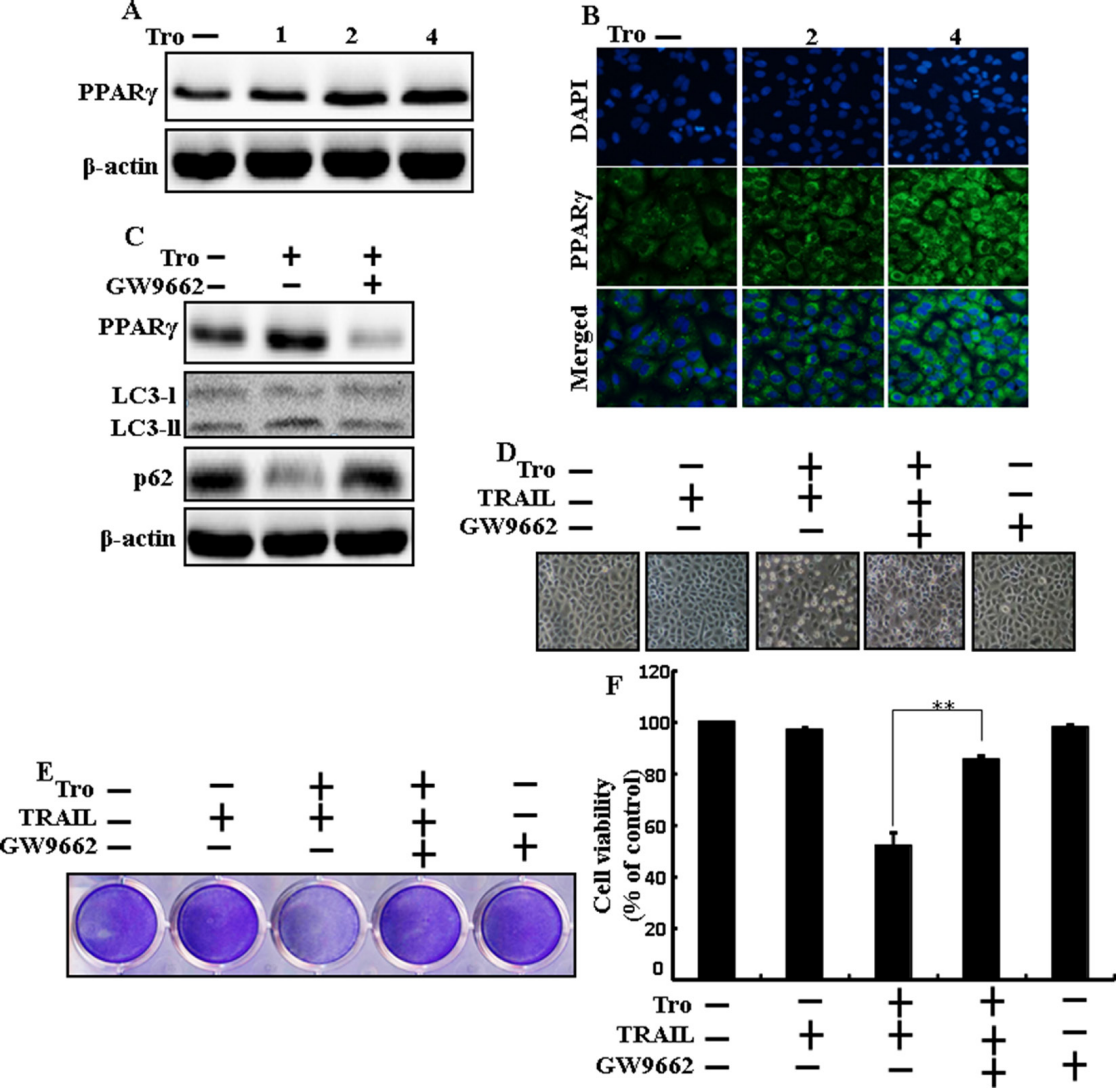
Effects of troglitazone are induced by PPARγ activation in A549 cells Lung adenocarcinoma cells were pre-incubated with different doses of troglitazone (0, 1, 2, and 4 μM) for 12 h and exposed to TRAIL protein for an additional 2 h. Additional cells were pretreated with GW9662 for 1 h followed by treatment with troglitazone. After that, (**A** and **C**) Western blot for PPARγ, LC3-II, and p62 proteins was analyzed from A549 cells; (**B**) Cells were immunostained with PPARγ antibody (green) and observed in fluorescent view; (**D**) Cell morphology photographed using light microscope (×100); (**E**) Cell viability was measured with crystal violet assay; (**F**) Bar graph indicating average density of crystal violet. β-actin was used as the loading control. **p < 0.01: represent significant differences between control and each treatment group; Tro: Troglitazone; TRAIL: Tumor necrosis factor (TNF)-related apoptosis-inducing ligand.

To understand the effects of troglitazone are induced by PPARγ activation in A549 cells. Pretreatment of troglitazone induced activation of PPARγ in a dose-dependent manner (Figure 7A and 7B). Western blot result demonstrated that the conversion rate of LC3-I to LC3-II and PPARγ was suppressed in the presence of GW9662 (Figure 7C). Morphological image and crystal violet staining results display that co-treatment with troglitazone, GW9662, and TRAIL enclosed the cell death effect compared to treatment with troglitazone and TRAIL (Figure 7D–7F). These data suggested that troglitazone-induced autophagy flux was dependent on the activation of PPARγ in A549 cells.

## DISCUSSION

The purpose of this study was to determine the effect of troglitazone with or without TRAIL on lung adenocarcinoma A549 cells. Our results demonstrated that PPARγ activation by troglitazone enhances TRAIL-mediated tumor cell death in A549 cells via regulation of autophagy flux.

TRAIL is a member of cytokine superfamily with the ability to selectively trigger cancer cell death but does not motive cytotoxicity to normal cells. The therapeutic probability of TRAIL is being demonstrated in various clinical trials [47]. The PPARγ ligands include naturally appearing fatty acids and members of the thiazolidinedione (TZD) group, such as troglitazone and rosiglitazone, and it has been reported extensively that PPARγ ligands inhibit cellular proliferation and selectively induce apoptosis [28, 48–54]. Antidiabetic drugs troglitazone and other TZDs have been investigated to suppress growth and to stimulate apoptosis and autophagy in a number of tumorigenic models and PPARγ has been recommended to be involved [55, 56]. Autophagy flux is the whole process through which cytoplasmic elements are initiated to lysosomes for degradation [57, 58]. Therefore, autophagy is outgoing as an important destination for the interruption and treatment of tumors [59], and targeting the autophagic pathways yields an innovative strategy for drug detection and new goals for drug improvement in cancer treatment [60].

Recent evidence has shown that considerable extent of cancer cell lines, including A549 cells were highly resistant to TRAIL-induced apoptosis [61]. In our present study, we also observed that Treatment of TRAIL or troglitazone alone did not or only slightly induced-apoptosis (Figure 1) and did not morphological change was identified compared with the control. We suggested that troglitazone and TRAIL co-treatment motives a significant induction of cancer cell death in lung A549 cells that are highly resistant to either agent alone. Some reports have demonstrated that troglitazone treatment inhibited cancer cell proliferation and induction of autophagy [37]. However, our western blot and immunocytochemistry results suggested LC3-II was increased and p62 was decreased after troglitazone treatment in a dose- dependent manner, though co-treatment of troglitazone with TRAIL enhanced intracellular apoptosis indicators Ac-cas3 and Ac-cas8 expression levels compared to treatment with troglitazone or TRAIL alone (Figure 2). Our results also suggested that specific pharmacological inhibitor chloroquine promoted the survival of lung adenocarcinoma A549 cells (Figure 3 and Figure 4). In addition, genetic autophagy inhibitor blocked troglitazone mediated apoptosis of A549 cells induced by TRAIL (Figure 5 and Figure 6). The ability of troglitazone to suppress a number of tumors has been attributed to its potential to inhibit growth and trigger apoptosis in a PPARγ-dependent manner [35, 62]. Recently revealed that PPARγ activation depend on autophagy, since GW9662 was able to prevent the upregulation of Beclin-1 as well as the accumulation of LC3 and MDC labeled vacuoles in breast cancer cells [63]. Our results demonstrated that Pretreatment of troglitazone induced activation of PPARγ in a dose-dependent manner. Western blot result suggested that the conversion rate of LC3-I to LC3-II and PPARγ was suppressed in the presence of GW9662 (Figure 7).

In summary, PPARγ activation by troglitazone sensitizes TRAIL-induced tumor cell death in A549 cells via autophagy flux. Combined treatment of troglitazone with TRAIL might be an adequate therapeutic technique to carefully treat some TRAIL-resistant cancers, including lung adenocarcinoma cells.

## MATERIALS AND METHODS

### Cell culture

Cancer cells originating from human lung (A549, HCC-15 and Calu-3) tumors were obtained from the American Type Culture Collection (Global Bioresource Center, Manassas, VA, USA). Cells were maintained in RPMI-1640 (Gibco BRL, Grand Island, NY, USA) medium containing 10% fetal bovine serum and 100 μg/ml penicillin-streptomycin. Cells were maintained at 37°C and 5% CO_2_ in humidified incubator.

### Reagents

Recombinant troglitazone, chloroquine (20 μM) and GW9662 (10 μM) were purchased from Sigma-Aldrich (St. Louis, MO, USA). Recombinant TRAIL (200 ng/ml) was purchased from Abfrontier (Geumcheon-gu, Seoul, South Korea).

### Cell viability assay

A549, HCC-15 and Calu-3 cells were plated at 1.0 × 10^4^ cells/well in 12-well plates and incubated at 37°C for 24 h. The A549 cells were pretreated with troglitazone in a dose-dependent manner (0, 1, 2, and 4 μM). After pretreated with different doses of troglitazone for 12 h and were treated with TRAIL protein for an additional 2 h. Additional cells were also pretreated with chloroquine (20 μM) and GW9662 (10 μM) for 1 h, followed by troglitazone treatment. Cell morphology was examined by photographs taken under inverted microscopy (Nikon, Japan). Cell viability was determined applying crystal violet staining method as previously described [64].

### Trypan blue exclusion assay

The number of cell viability was examined by trypan blue dye exclusion assay (Sigma-Aldrich) using a hemocytometer. The result was mainly expressed as the percentage of viable cells compared with that of vehicle-treated controls.

### Western blot assay

A549 cell lysates were prepared by harvesting, washing in cold PBS, resuspending in lysis buffer followed by sonication. Proteins (35 μg) were resolved by 10%–15% SDS gels and transferred to a nitrocellulose membrane, and analyzed by western blotting as described previously [65]. The following antibodies were used for immunoblotting: LC3(Novus Biologicals, Littleton, CO, USA), DR-4, DR-5, and β-actin Sigma-Aldrich (St. Louis, MO, USA), p62 (Millipore Corp., Milford, MA, USA), ATG5, cleaved caspase-3 (Cell Signaling Technology, Danvers, MA, USA), cleaved caspase-8 (BD pharmingen, USA), PPARγ Santa Cruz Biotechnology, Inc. (Santa Cruz, CA, USA).

### Immunocytochemistry

A549 cell lines cultured on glass coverslips positioned on a 24-well plate. The cells were washed with PBS and fixed with 4% paraformaldehyde for 15 min at room temperature. Following this, Cells were then washed twice with ice-cold PBS, blocked with 5% FBS inTris-buffered saline with Tween, and incubated with monoclonal antibodies against p62, PPARγ at room temperature for 24 h. Unbound antibody was removed with PBS wash ( three times) and Cells were then incubated again with secondary antibody at room temperature for 2 h in the dark. Finally, cells were mounted with DakoCytomation fluorescent mounting medium and visualized via a fluorescence microscopy.

### TEM (transmission electron microscopy) analysis

TEM samples were analyzed by Transmission Electron Microscope (JEM-2010, JEOL) installed in the Center for University-Wide Research Facilities (CURF) at Chonbuk National University. After fixation of A549 cell samples in 2% glutaraldehyde and 2% paraformaldehyde in 0.05 sodium cacodylate buffer, specimens were post fixed in 1% osmium tetroxide, dehydrated in graded ethanol and propylene oxide. A549 cells were embedded in Epoxy resin. Ultrathin sections were cut on an LKB-III ultratome and were stained with 0.5% uranyl acetate and lead citrate. The images were taken on a Hitachi H7650 electron microscope at an accelerating voltage of 100 kV.

### RNA interference

A549 cells were transfected with ATG5-specific small interfering RNA (siRNA; oligo ID HSS114103; Invitrogen, Carlsbad, CA, USA) using Lipofectamine 2000 according to the manufacturer's instructions. After 36-h post transfection, the knockdown efficiency at protein level was observed by immunoblotting and cell viability test. Nonspecific siRNA was used as a negative control.

### Statistical analysis

All data are expressed as means ± standard deviation (SD) and were compared using the Student's *t*-test, analysis of variance and the ANOVA Duncan test using SAS statistical package (SAS Institute, Cary, NC, USA). Statistical significance was indicated by a *P* value less than 0.05 (*), 0.01 (**), or 0.001 (***).
